# Metabolic Network-Based Identification and Prioritization of Anticancer Targets Based on Expression Data in Hepatocellular Carcinoma

**DOI:** 10.3389/fphys.2018.00916

**Published:** 2018-07-17

**Authors:** Gholamreza Bidkhori, Rui Benfeitas, Ezgi Elmas, Meisam Naeimi Kararoudi, Muhammad Arif, Mathias Uhlen, Jens Nielsen, Adil Mardinoglu

**Affiliations:** ^1^Science for Life Laboratory, KTH—Royal Institute of Technology, Stockholm, Sweden; ^2^Department of Biology and Biological Engineering, Chalmers University of Technology, Gothenburg, Sweden

**Keywords:** hepatocellular carcinoma, genome-scale metabolic model, network analysis, biological networks, cancer, gene expression, protein expression, systems biology and network biology

## Abstract

Hepatocellular carcinoma (HCC) is a deadly form of liver cancer with high mortality worldwide. Unfortunately, the large heterogeneity of this disease makes it difficult to develop effective treatment strategies. Cellular network analyses have been employed to study heterogeneity in cancer, and to identify potential therapeutic targets. However, the existing approaches do not consider metabolic growth requirements, i.e., biological network functionality, to rank candidate targets while preventing toxicity to non-cancerous tissues. Here, we developed an algorithm to overcome these issues based on integration of gene expression data, genome-scale metabolic models, network controllability, and dispensability, as well as toxicity analysis. This method thus predicts and ranks potential anticancer non-toxic controlling metabolite and gene targets. Our algorithm encompasses both objective-driven and—independent tasks, and uses network topology to finally rank the predicted therapeutic targets. We employed this algorithm to the analysis of transcriptomic data for 50 HCC patients with both cancerous and non-cancerous samples. We identified several potential targets that would prevent cell growth, including 74 anticancer metabolites, and 3 gene targets (PRKACA, PGS1, and CRLS1). The predicted anticancer metabolites showed good agreement with existing FDA-approved cancer drugs, and the 3 genes were experimentally validated by performing experiments in HepG2 and Hep3B liver cancer cell lines. Our observations indicate that our novel approach successfully identifies therapeutic targets for effective treatment of cancer. This approach may also be applied to any cancer type that has tumor and non-tumor gene or protein expression data.

## Introduction

Hepatocellular carcinoma (HCC) is a primary form of liver cancer and one of the main causes of cancer mortality globally (Ferlay et al., 2015). Patient prognosis is usually poor and most result in patient death (Altekruse et al., 2009). This high mortality is partly due to the high tumor heterogeneity of this cancer (Friemel et al., 2015; Benfeitas et al., 2017), which makes it difficult to identify suitable and effective therapeutic targets. It is therefore urgent to devise suitable approaches capable of capturing the high tumor heterogeneity for identifying effective therapeutic targets for this cancer.

Biological network analysis is emerging as an efficient and suitable *in silico* approach to identify cellular targets for disease treatment incorporating cellular heterogeneity (Barabasi et al., 2011). For instance, network topology features such as centralized protein hubs have been employed in the analysis of protein-protein interaction (PPI) networks to identify potential therapeutic targets (Guney et al., 2016; Lv et al., 2016). A few efforts have applied network controllability to identify minimum sets of driver proteins for controlling PPI networks (Liu et al., 2011; Yuan et al., 2013), or indispensable proteins from a network controllability perspective (Vinayagam et al., 2016). Central and highly connected proteins and genes appear in bottleneck interactions (Wuchty, 2014), and tend to be essential from a lethality perspective (Jeong et al., 2001; Yu et al., 2008; Najafi et al., 2014). However, these approaches have a limited scope in their considered essentiality analyses and do not take into account comprehensive descriptions of biological functionality, or the stoichiometry of the interactions.

Genome-scale metabolic models (GEMs) are whole cell stoichiometric representations of metabolism that take into account network functionality through prediction of a model's capacity to achieve one or more biological tasks (e.g., biomolecule synthesis and growth) (Mardinoglu and Nielsen, 2012, 2015; Mardinoglu et al., 2013). GEMs have been employed to identify essential genes or anticancer metabolites, build tissue-specific cellular characterizations, and explain cell behavior at the metabolic, signaling, gene, and protein level (Folger et al., 2011; Agren et al., 2014; Mardinoglu et al., 2014a,b; Varemo et al., 2015). However, GEM-based methods often do not prioritize between different candidate therapeutic targets, and assessing toxicity to normal tissues is not always possible if the essential nodes are not contemplated by the flux distribution. Further, these approaches often do not allow for ranking between different candidate targets.

Here, we overcome the limitations of current state-of-the-art methods by introducing a network-based prioritization approach to identify and prioritize non-toxic metabolic targets for disease treatment using network controllability, topology analysis, and constraint-based modeling techniques. Our algorithm approach permits analyzing cellular network behavior using any kind of expression data such as microarray, transcriptomics (RNA-seq), or protein expression. Briefly, this algorithm combines GEMs, personalized metabolite-metabolite and reaction-reaction association networks. Based on the analysis of these networks, it determines metabolites/genes whose perturbation has a strong effect on network dynamics (i.e., minimum driver set nodes) (Yuan et al., 2013), indispensable metabolites/genes (Vinayagam et al., 2016), and several network topology parameters (i.e., node centrality analysis). Based on this information, this algorithm determines and ranks anticancer metabolites/genes, excluding those that would also become toxic for non-cancerous tissues.

We employed this algorithm in the analysis of RNA-seq data of hepatocellular carcinoma (HCC), the most prevalent form of liver cancer and third leading cause of cancer-related mortality worldwide (Ferlay et al., 2010). Through integration of transcriptomic data from cancerous and non-cancerous samples and personalized systems biology approaches, we identified cancer-specific targets. These targets were experimentally validated in HepG2 and Hep3B cells, cell lines frequently used as models for liver cancer.

## Methods

### Algorithm overview and requirements

The algorithm presented here requires Matlab R2016a, RAVEN and tINIT (Agren et al., 2013, 2014) and has been implemented on a Dell laptop running Windows 7. It comprises the 4 major steps detailed below: 1. Objective-dependent identification of potential targets; 2. Creation of personalized metabolic and reaction networks; 3. Controllability analysis; and 4. Target prioritization. We provide an explanation for each of these steps below, as well as pseudo-code or original references showing their implementation.

### Step 1. objective-dependent identification of potential targets

We identify metabolites and genes that potentially serve as therapeutic targets based on antimetabolites and *in silico* gene silencing of personalized GEMs. The two approaches rely on objective function and metabolic tasks for cancer and non-cancerous tissues to determine the functional outcome, i.e., viable vs. non-viable. Viability is determined for instance by testing whether objective-functions are non-null. Importantly, toxicity testing is done by identifying those antimetabolites/silenced genes that render tumors non-viable, but maintain viability in non-cancerous cells.

Step 1a. Antimetabolites are those structurally similar to endogenous metabolites and that prevent cell growth. Basically, for each metabolite and regardless of compartmentalization, an antimetabolite is identified by removing the reactions where the metabolite serves as substrate. This step was implemented using tINIT as previously indicated (Agren et al., 2014).

Step 1b. The concept of *in silico* gene silencing is similar to lethality analyses but considers metabolic tasks (Supplementary File 1) in addition to objective functions to assess model functionality, to assess the effect of gene silencing. In order to simulate the effect of gene silencing for each gene on the models, we removed the reaction(s) catalyzed by the gene.

**Algorithm STEP 1b:**
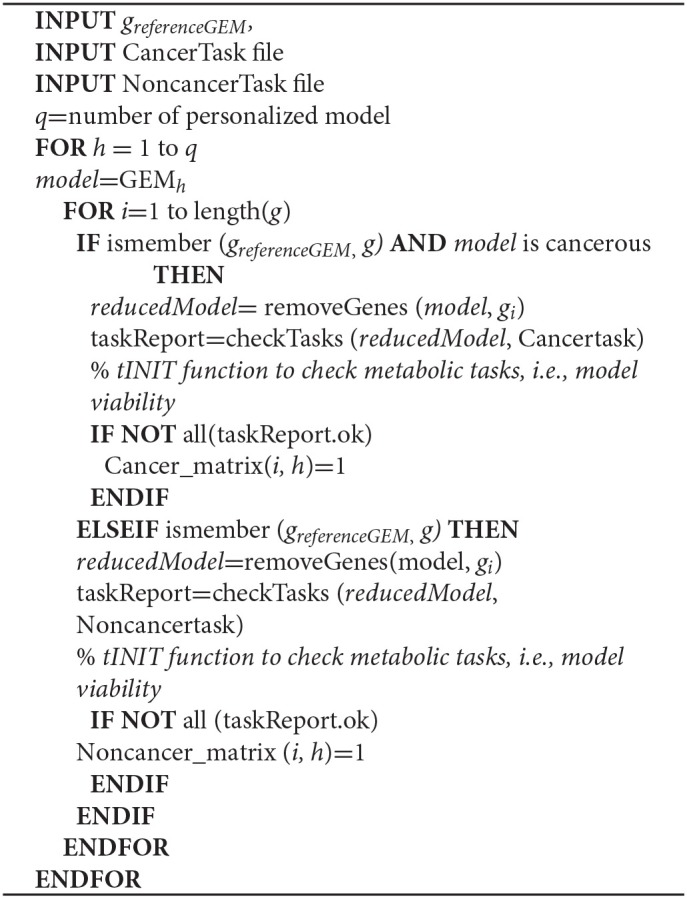
*In silico* gene silencing

Step 1c. The predicted antimetabolites (Step 1a) and silenced genes (Step 1b) are filtered to select only those found in HCC GEMs but not in non-cancerous GEMs.

### Step 2. creation of personalized metabolic and reaction networks

Personalized GEMs are used to generate directed Metabolite-Metabolite network (MMN, Step 2a), and directed Reaction-Reaction network (RRN, Step 2b). MMN and RRN are simple graphs *G*(*V, E*) with *V* nodes and *E* edges. In MMN, nodes (metabolites) are connected by an edge if they are involved in the same reaction. In RRN, nodes (reactions) are connected if the product of a reaction serves as substrate by the other (Supplementary Figure 1).

### Step 3. controllability analysis

We identify minimum driver set nodes (MDS), those responsible for controlling network dynamics upon perturbation (Yuan et al., 2013), as well as indispensable nodes (Vinayagam et al., 2016), from MMN (Step 2a) and RRN (Step 2b). MDS and indispensable nodes (metabolites or genes) are jointly used for subsequent steps, and termed *controlling nodes*.

Step 3a. We identify MDS in MMN and RRN according to the Popov–Belevitch–Hautus (PBH) rank condition (Sontag, 2013; Yuan et al., 2013).

**Algorithm STEP 2a:**
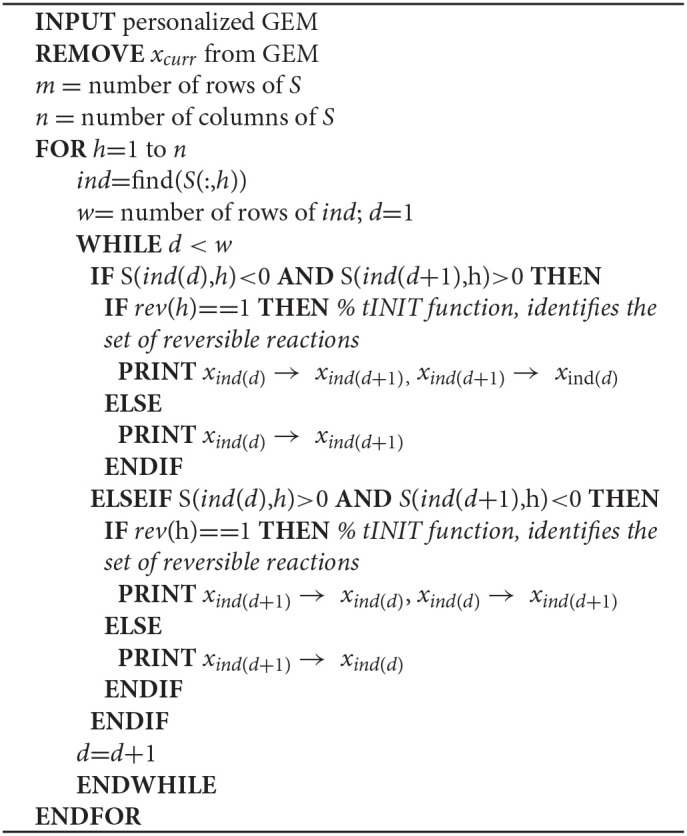
Creation of directed metabolite-metabolite networks

Step 3b. Node dispensability is determined by assessing the maximum geometric multiplicity of the eigenvalue λ*m* upon removing each node.

Step 3c. We excluded those nodes that were simultaneously identified in both HCC and non-cancerous networks in Steps 3a and 3b.

### Step 4. target prioritization

In the final step, we filter nodes by selecting those with anticancer properties (Step 1, metabolic task dependent) that are also controlling metabolites/genes (Step 3, metabolic task independent). This generates a list of *anticancer non-toxic controlling nodes* that are then ranked based on their centrality properties.

Step 4a. Filter nodes simultaneously identified as controlling nodes (Step 3) with anticancer properties (Step 1).

Step 4b. We compute topological parameters for MMN and RRN. Specifically, we compute node degree and betweenness centrality using default commands [e.g., Matlab R2016a built-in *degree()* and *centrality()* functions].

Step 4c. Based on each node's topological measures, we rank anticancer controlling nodes. Indispensable nodes are ranked based on degree centrality whereas MDS are ranked based on betweenness centrality. This results in a list of nodes (metabolites/genes) with anticancer and controlling properties, sorted according to their topological importance in the networks.

**Algorithm STEP 2b:**
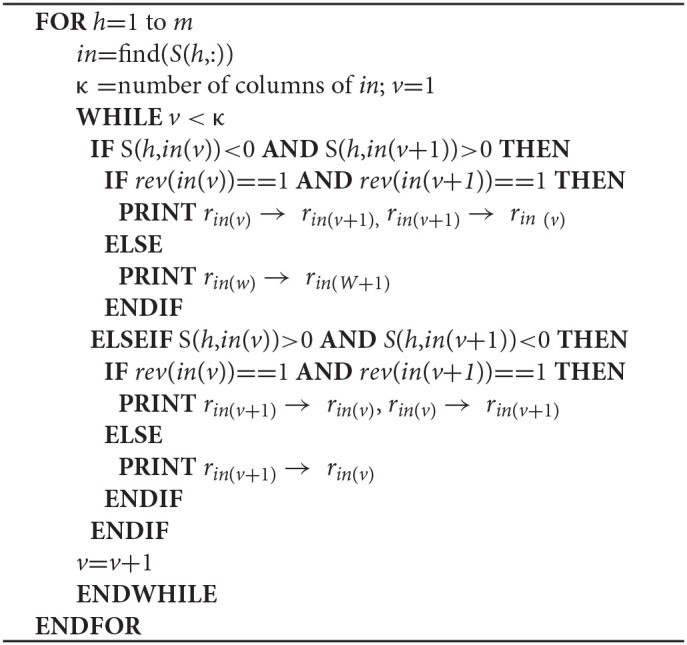
Creation of directed reaction-reaction networks

### Gene expression data and modeling for HCC patients

Transcriptomic data (RNA-seq) for 50 HCC and 50 adjacent non-cancerous liver samples were retrieved from NCI's Genome Data Commons (Grossman et al., 2016) as Fragments Per Kilobase of transcript per Million mapped reads (FPKM). Personalized GEMs were built by integrating transcriptomic data with the liver-specific GEMs through tINIT and RAVEN (Agren et al., 2013, 2014) using the reference human GEM HMR2 (Uhlen et al., 2017).

The following thresholds for gene levels were considered: no expression (FPKM < 1), low expression (1 ≤ FPKM < 10), medium expression (10 ≤ FPKM < 50), and high (FPKM≥50). We considered biomass production or ATP consumption as objective functions for HCC and non-cancer GEMs, respectively. Model functionality of HCC and non-cancer GEMs was respectively determined based on 57 and 56 metabolic tasks, as previously determined (Agren et al., 2014). Cell growth is considered as an additional metabolic task for HCC.

### HepG2 and Hep3B cell line experiments

HepG2 and Hep3B, human hepatocellular carcinoma cell lines frequently used as models for liver cancer (Qiu et al., 2015), were cultured in RPMI-1640 Medium (R2405, Sigma-Aldrich) and Minimum Essential Medium Eagle (M4655, Sigma-Aldrich), respectively. Both cell lines were supplemented with 10% fetal bovine serum (FBS, F2442, Sigma-Aldrich) and incubated in 5% CO_2_ humidity at 37°C. To confirm the effect of silencing of CRLS1, PRKACA and PGS1 genes on cancer cell growth and viability, we used RNA interference (RNAi) to inhibit gene expression. Cells were infected by predesigned targeted Silencer® siRNAs for each gene (Thermo Fisher Scientific, clone IDs shown in Supplementary File 2), at 35 nM by using Lipofectamine® RNAiMAX (13778075, Life Technologies). Cells incubated in medium with non-targeting negative control siRNA at 35 nM (4390843, Life Technologies) were assigned as control.

**Algorithm STEP 3b:**
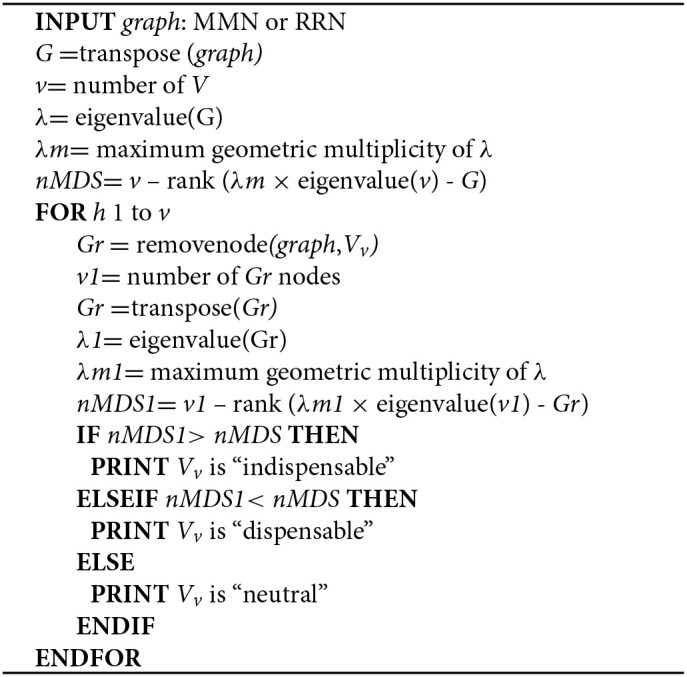
Determination of node dispensaiblity

Total RNA was isolated with RNeasy mini Kit (15596026, Thermo Fisher Scientific) after treatment with siRNA for 24 h. The expression profiles of key genes (CRLS1, PRKACA and PGS1) in HepG2 and Hep3B cells separately were measured and analyzed via quantitative real-time PCR with iTaq Universal SYBR Green One-Step Kit (1725151, Bio-Rad), using anchored oligo (dT) primer based on CFX96™ detection system (Bio-Rad). GAPDH was set as the internal control for normalization. Primer sequences are listed in Supplementary File 2. Cell Counting Kit-8 (CCK-8, Sigma-Aldrich) was used to detect variation in cell proliferation after interference by siRNA for 24 h.

All experiments were performed strictly according to the manufacturer's instructions and repeated in triplicate.

### Statistics

Statistic comparison between siRNA-targeted gene expression and non-negative controls was performed using Welch's *t*-test, corrected for Benjamini & Hochberg.

## Results

### Algorithm

GEMs include metabolites (*x*), reversible and irreversible reactions (*r*), and associated genes (*g*). Reactions are represented as a stoichiometric matrix (*S*) of size *m* × *n*, where rows (*m*) in *S* represent metabolites and columns (*n*) correspond to reactions in the model (Supplementary Figure 1). Each entry is a stoichiometric coefficient *s*_*ij*_ of the metabolites that participate in a reaction. *s*_*ij*_ ≥ 1 and *s*_*ij*_ ≤ −1 respectively indicate that metabolite *i* is a product or reactant in reaction *j*, whereas *s*_*ij*_ = 0 indicates no involvement in the reaction. Reaction reversibility is discriminated by the *rev* vector, where 0 and 1 respectively indicate an irreversible or reversible reaction. Currency metabolites (*x*_*curr*_) such as cofactors, coenzymes and H_2_O are found in a number of reactions and considered by the GEMs, but are disregarded when creating metabolite-metabolite and reaction-reaction association networks(Khosraviani et al., 2016).

**Figure 1:**
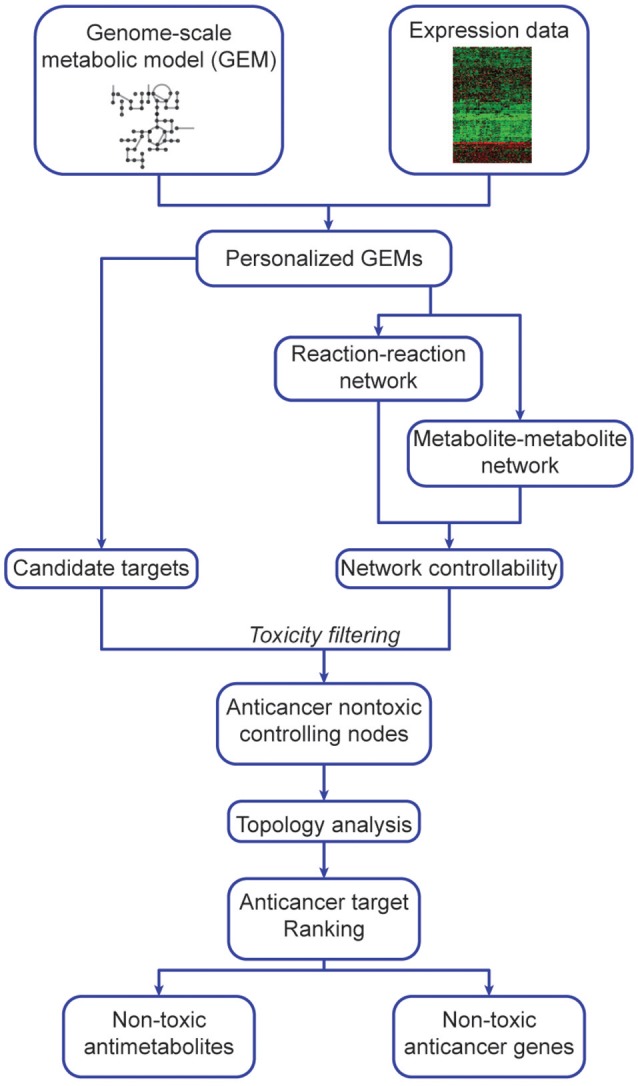
Outline of network-based identification and prioritization of metabolite and gene anticancer targets.

Our algorithm (Figure 1, Supplementary Figure 2) predicts potential metabolite and gene targets that display anticancer activity but are non-toxic to non-cancerous tissues. To do so, it integrates expression data (transcriptomics, microarray or protein levels) into GEMs (Methods, Algorithm steps 1b–2b) to generate cancer and non-cancer specific models. After network manipulation and analysis, it identifies and prioritizes anticancer metabolites and gene targets (Algorithm step 3b). These were implemented in Matlab, and require RAVEN and tINIT (Agren et al., 2013, 2014). Our hypothesis follows the determination of metabolite or genes that display anticancer and network-controlling properties, and sorting them according to their topology parameters. The highest ranking (most central) nodes are potentially better targets due to their pivotal and central role in network dynamics, and are excluded if identified in non-cancerous tissues to prevent toxicity. Several filtering steps are taken to exclude false positives: candidate anticancer targets must have network controlling properties and cannot be found in non-cancerous networks, i.e., they must be anticancer non-toxic controlling nodes.

This algorithm consists of 4 main steps, applied to both cancer and non-cancer personalized GEMs (details in section Methods), constructed using expression data and reference models (Uhlen et al., 2017). Step 1 is independent of steps 2 and 3, and all are integrated at Step 4.

1. Objective-dependent identification of potential targets
  - Antimetabolite analysis
  - *In silico* gene silencing
  - Toxicity analysis
2. Creation of personalized metabolite and reaction networks
  - Creating metabolite-metabolite networks
  - Creating reaction-reaction networks
3. Controllability analysis
  - Identification of Minimum Driver Set Nodes
  - Dispensability analysis
  - Toxicity analysis
4. Target prioritization
  - Determining anticancer non-toxic controlling nodes
  - Computation of centrality parameters
  - Target prioritization

All steps are described in detail according to a toy model (Supplementary Figures 1, 2) and pseudo-code is described in section Methods.

### Determination of antimetabolites, anticancer genes, and networks based on personalized GEMs in HCC

As a test case, we sought to identify non-toxic cancer targets using 50 HCC and 50 non-cancerous transcriptomic samples from TCGA. This test case employs liver-specific GEMs (Uhlen et al., 2017), which encompass 7,780 reactions and 2,857 metabolites controlled by 2,892 genes. We built functional personalized GEMs for HCC and non-cancerous samples (Supplementary File 3) where model functionality was determined based on the metabolic tasks and the objective functions.

**Figure 2:**
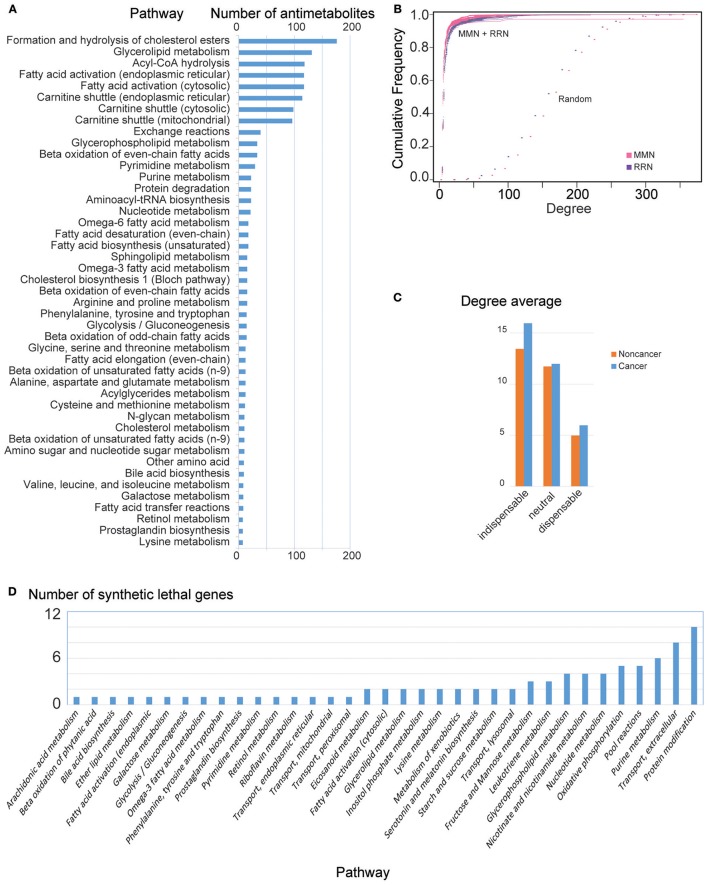
General MMN and RRN node features in HCC. **(A)** Number of antimetabolites identified per pathway. Note that a predicted antimetabolite may be found in multiple pathways. **(B)** Cumulative degree distribution for MMN (purple) and RRN (magenta), compared to randomly generated models (Erdos–Rényi, dots). RRNs show qualitatively similar properties (results not shown). **(C)** Mean degree for indispensable, neutral, and dispensable metabolites in noncancer and HCC MMNs. RRNs show qualitatively similar properties (results not shown). **(D)** Number of genes leading to lethality identified per pathway. Note that a predicted synthetic lethal gene may be found in multiple pathways.

We then identified 374 antimetabolites out of 2,857 metabolites that inhibited growth of HCC GEMs, but passed all metabolic tasks in non-cancerous GEMs, i.e., *non-toxic* cancer-specific antimetabolites (Steps 1a and 1c, Supplementary File 4). Most antimetabolites are involved in carnitine shuttle, fatty acid activation, and acyl-CoA hydrolysis, as well as metabolism of cholesterol, glycerolipid, glycan, purine, pyrimidine, nucleotide, amino acid, and proteins (Figure 2A).

Based on *in silico* gene silencing, we found that 2,204 genes did not fulfill all 56 metabolic tasks in non-cancerous models. We identified 283 genes that inhibited the growth in at least one HCC, and 8 genes that inhibit growth in all HCC models, but induced no change in non-cancer GEMs (Steps 1b and 1c, Figure 2D, Supplementary File 4).

We also generated MMN and RRN for each personalized GEM (Steps 2a and 2b, respectively). The generated MMN and RRN showed scale free (power-law) degree distribution, and the cumulative degree distribution of the networks were compared with random networks generated by Erdos–Rényi model (Figure 2B).

### Identification of non-toxic anticancer controlling metabolites using metabolite-metabolite network controllability

We identified cancer-exclusive controlling metabolites in all GEMs (Step 3, Supplementary File 4). Specifically, we found 201 MDS metabolites, most of which are involved in exchange and transporting reactions. Most amino acids, (deoxy)nucleotides, fatty acids and cholesterol, and some sugars including mannose, glucose, fructose, and galactose, are MDS in HCC models. In turn, we identify 578 indispensable metabolites, most of which either start metabolic pathways or are important metabolites in the networks, such as malonyl-CoA, palmitoyl-CoA, glucose, mannose, glucose-6-phosphate, HMG-CoA, (deoxy)nucleotides, DNA, acetyl-CoA, amino acids, L-carnitine, fatty acids, and coenzymes. Indispensable metabolites present higher degree in MMN (Supplementary File 4), in comparison with other node types in both non-cancer and cancer networks (Figure 2C). Because of this, the indispensable nodes are ranked based on node degree parameters (Step 4c). After removing metabolites simultaneously found in HCC and non-cancerous networks, we identify 142 cancer-specific controlling metabolites which were used in the subsequent steps (Supplementary File 4). The 374 antimetabolites found in Steps 1a and 1c were filtered based on the 142 cancer-specific controlling metabolites. This results in 74 non-toxic anticancer metabolites with network controlling properties, potential targets for antimetabolite treatment (Step 4a). Among the 74 anticancer controlling metabolites, 54 are indispensable and 20 are MDS (Supplementary File 4). The anticancer indispensable controlling metabolites shows substantially higher degree (Supplementary File 4). For instance, the top ranking indispensable antimetabolites show high degree (leucine 79, isoleucine 78, UMP 66, malonyl-CoA 34, palmitoyl-CoA 16) when compared with mean metabolite degree = 6. The 75 anticancer controlling metabolites are mostly involved in metabolism of DNA and nucleotides, amino acids, and fatty acids.

Importantly, while several nodes present very high centrality, they may not be suitable therapeutic targets because their silencing either does not lead to lethality, or leads to lethality in both cancer and non-cancer networks (i.e., toxicity, Supplementary Files 4, 5).

Overall, our network-based approaches highlight several potential metabolic targets for cancer-specific treatment.

### Identification of gene targets exclusive to HCC using reaction-reaction network and experimental validation

**Figure 3:**
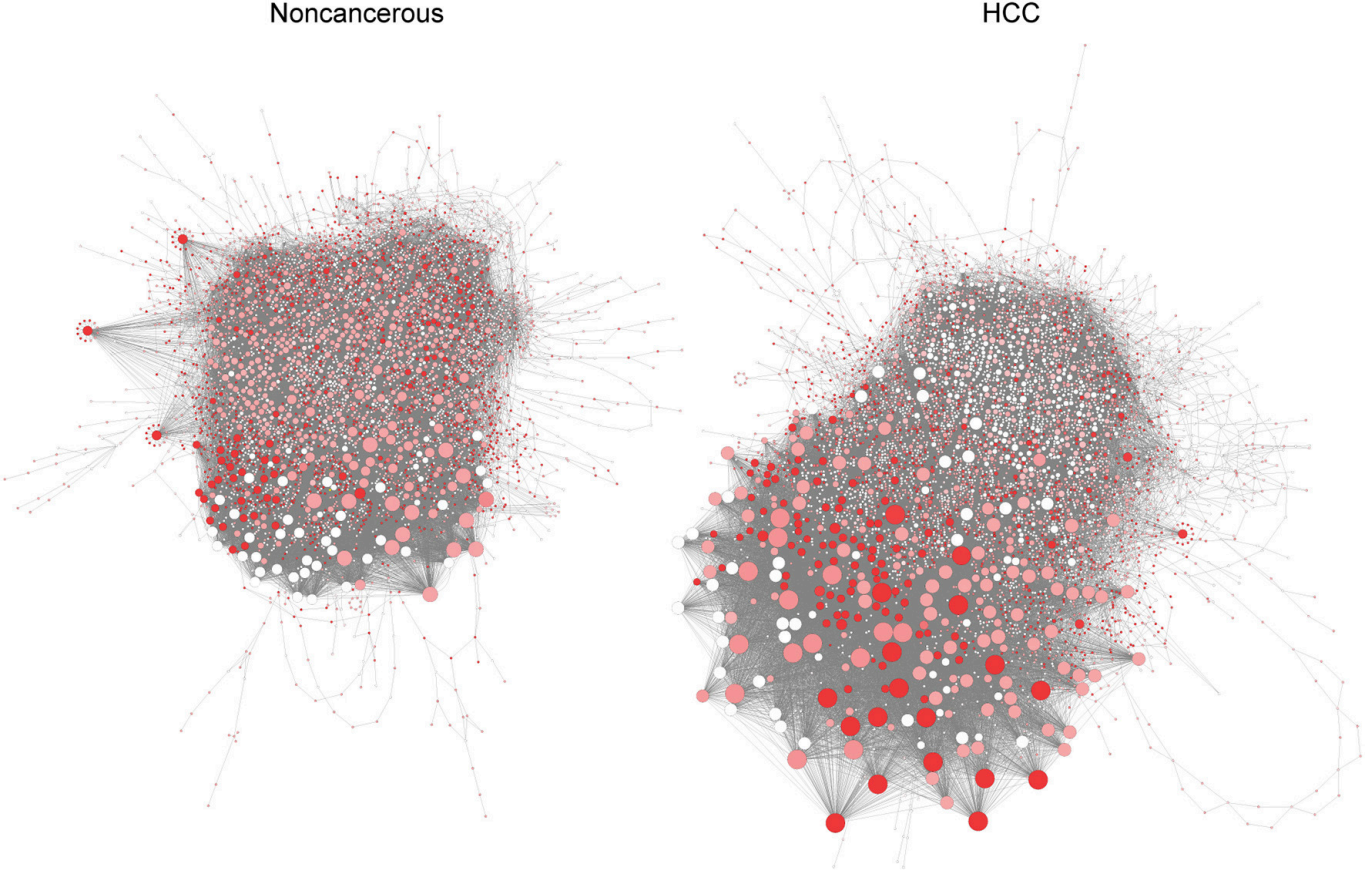
Pivotal genes in HCC **(Left)** and non-cancerous **(Right)** RRN. Node size is proportional to degree centrality, and controlling anticancer genes are highlighted (red means higher number of patients where gene silencing leads to lethality and where a gene is a controlling node).

Silencing of controlling genes in RRN shows specific features in HCC and non-cancerous networks (Figure 3). Among the eight genes that inhibit growth in all HCC but not in non-cancerous models, we identify three genes that are controlling nodes in HCC RRN but not in non-cancerous RRN (Step 3, Supplementary File 5): PRKACA (protein kinase cAMP-activated catalytic subunit alpha), PGS1 (phosphatidylglycerophosphate synthase 1), CRLS1 (cardiolipin synthase 1). Since they are indispensable nodes, we then ranked them based on degree parameter as PRKACA, PGS1, and CRLS1 (Supplementary File 5), from most to least central genes.

**Figure 4:**
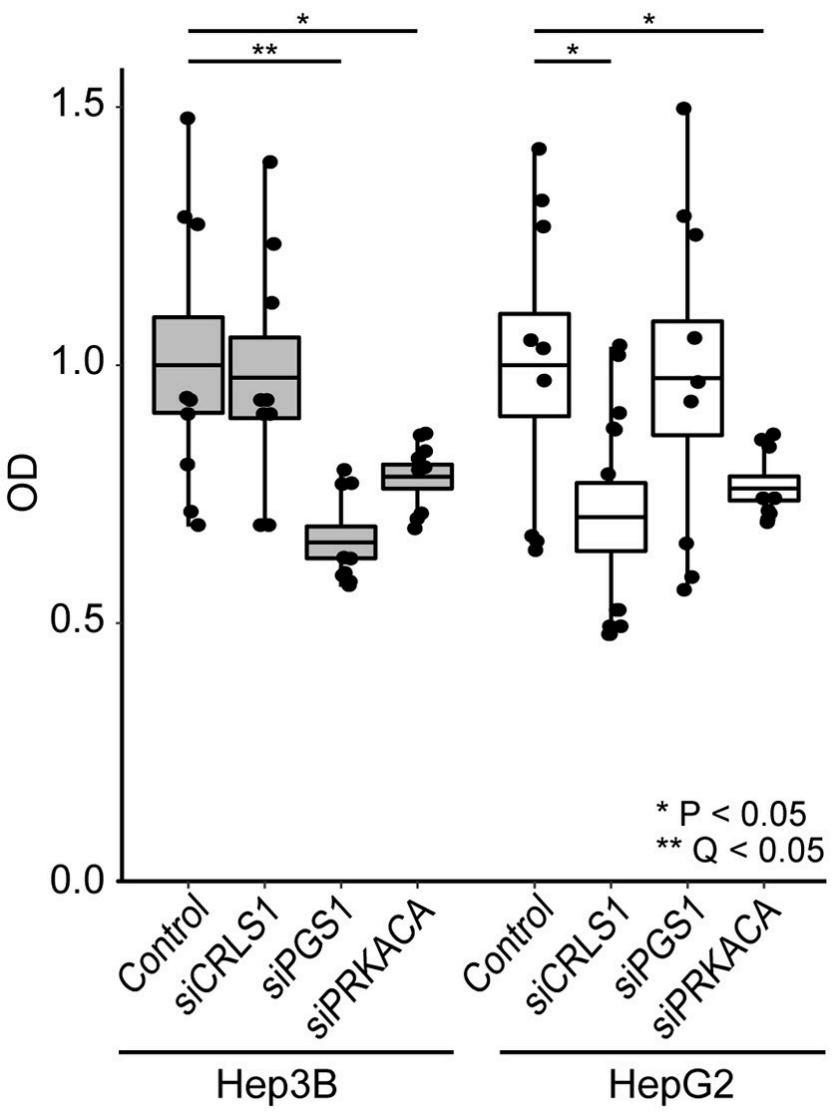
siRNA inhibition of CRLS1, PGS1, and PRKACA expression in Hep3B and HepG2 liver cell lines leads to decreased growth after 24 h in comparison with controls. Comparisons performed using Welch's *T*-test. See methods for details.

We tested the anticancer properties of these three genes using siRNA in HepG2 and Hep3B cells (Figure 4). Cell viability was tested 24 h after gene silencing using the CCK-8 assay (see Methods). As negative controls, cells were exposed to a non-targeting siRNA. Our observations indicate that PRKACA silencing led to 22–24% decrease in cell growth in both cell lines (*P* < 0.05). CRLS1 silencing led to 30% lower growth in HepG2 (*P* < 0.05) but no change in Hep3B. Finally, PGS1 silencing produced the most substantial growth change, decreasing growth in Hep3B by 35% (*Q* < 0.05).

## Discussion

Network analyses have been extensively used in understanding cellular response to disease and predicting targets for treatment. Due to their comprehensive and integrative nature, networks successfully and efficiently capture multidimensional biological heterogeneity (Mardinoglu and Nielsen, 2012, 2015; Mardinoglu et al., 2013; Najafi et al., 2014; Benfeitas et al., 2017). Such cases include topology (Asgari et al., 2013), controllability (Liu et al., 2011; Yuan et al., 2013; Wuchty, 2014; Kanhaiya et al., 2017), dispensability (Vinayagam et al., 2016), and genome-scale metabolic models (Mardinoglu and Nielsen, 2012, 2015; Mardinoglu et al., 2013; Agren et al., 2014) in prediction of disease targets. For instance, a recent analysis shows that a small number of proteins essential for survival are controlled by a small set of driver nodes (Kanhaiya et al., 2017). Network controllability analyses have also highlighted that highly connected proteins are critical for cell survival (Jeong et al., 2001; Jonsson and Bates, 2006). In turn, others have used metabolic networks to identify candidate metabolite analogs and genes with anticancer properties (Agren et al., 2014). While these efforts have undoubtedly provided great contributions to clarify the underlying disease biological phenomena, and identified pivotal components for controlling network dynamics, such methods have specific shortcomings. For instance, topology-based approaches often do not take into account interaction stoichiometry, and essentiality analyses do not consider multiple functionality properties necessary for growth, e.g., energetic and redox balance, maintenance of central metabolism (see Agren et al., 2014 and references therein).

Here, we developed an algorithm that integrates expression data with both *objective-dependent* and *objective-independent* approaches to identify and rank metabolites and genes for cancer treatment. Objective dependent approaches such as flux balance analysis, employed here, base their predictions on satisfying specific biological phenomena. However, our method overcomes some of the disadvantages in FBA-based approaches which, unlike our method, do not permit ranking multiple candidate targets, and assessing potential toxic targets is not always possible if the essential nodes are not contemplated by the flux distribution. Objective independent approaches, including network topology, controllability, and dispensability analysis employed here, permit classifying and ranking between nodes. In our algorithms, candidate targets are filtered at multiple steps based on their toxicity, network controllability and anticancer properties. Thus, and unlike typical network topology analysis, our proposed method incorporates biological functionality expressed as metabolic requirements, and reaction stoichiometry. To our knowledge, this is the first such case where prioritization and classification of potential metabolite/gene targets is determined while considering toxicity to non-cancerous tissues and biological functionality.

We employed this algorithm for the analysis of hepatocellular carcinoma, a deadly form of liver cancer (Ferlay et al., 2010). This transcriptomic dataset comprised 50 tumor and 50 non-cancerous samples, retrieved form TCGA (Weinstein et al., 2013). We identify 142 metabolites, and 3 genes as potential therapeutic targets for non-toxic treatment of this liver cancer.

The non-toxic anticancer metabolites identified in here tend to be involved in metabolism of DNA and nucleotides, amino acids, and fatty acids. Several observations support utilization of analogs of these metabolites or targeting these biological processes for cancer treatment. For instance, several pyrimidine or purine analogs are among FDA-approved drugs for cancer therapy in DrugBank (Law et al., 2014), such as Gemcitabine, Fluorouracil, Clofarabine, Azacitidine, Floxuridine, Cladribine, Raltitrexed, Tioguanine, Azacitidine. All amino acids analogs were found as antimetabolites but most of them were filtered based on cancer controlling metabolites as toxic targets based on controllability analysis. Among all amino acids, glutamine, leucine, and isoleucine were introduced as nontoxic anticancer metabolite targets. Several studies show the critical role of amino acids such as glutamine in cancer (Xie et al., 2012; Bhutia et al., 2015; Xiao et al., 2016; Fung and Chan, 2017; Maddocks et al., 2017). For instance, leucine and isoleucine supplementation promote tumorigenesis in rat bladder cancer (Xie et al., 2012), and leucine deprivation inhibits breast cancer proliferation in humans (Xiao et al., 2016).

Other metabolites such as fatty acids also display a crucial role in cancer. For instance, malonyl-CoA serves as substrate for fatty acid synthase, and inhibition of this enzyme with the synthetic inhibitor C75 (Kuhajda et al., 2000) significantly decreases growth in HepG2 cells (Gao et al., 2006). Our previous work identified L-carnitine and metabolites involved in L-carnitine biosynthesis as non-toxic anticancer HCC targets using protein levels for 6 HCC samples (Agren et al., 2014). Our algorithm presented here improves on this work by showing that L-carnitine is a non-toxic anticancer target for HCC treatment (Step 2, Supplementary File 3) using a different technology and sample, and network topology analyses indicate that other metabolites may have better network controlling properties than L-carnitine.

At the gene level, only 3 genes pass all the filtering criteria, and show anticancer non-toxic controlling properties: protein kinase cAMP-activated catalytic subunit alpha (PRKACA), phosphatidylglycerophosphate synthase 1 (PGS1), and cardiolipin synthase 1 (CRLS1). We experimentally confirmed that the 3 genes display anticancer properties using siRNA in HepG2 and Hep3B liver cancer cell lines. We observe that depletion of PGS1 and PRKACA greatly decreases growth in both HepG2 and Hep3B, whereas depletion of CRLS1 decreases expression in HepG2. These genes have been associated with alterations in proliferation, malignancy and apoptosis in several cancers. The cAMP-dependent protein kinase A has crucial signaling roles in cancer, promotes resistance to therapy and its inhibition blocks tumor invasion (Sabbisetti et al., 2005; Honeyman et al., 2014; Moody et al., 2015). PGS1 metabolizes phosphatidylglycerol in lipid metabolism, is involved in cellular transformation, and its upregulated in metastases (Hirsch et al., 2010; Hartung et al., 2017). Finally, cardiolipin synthase 1 is involved in phosphatidylglycerol metabolism, and alterations in cardiolipin metabolism are associated with proliferation and cell death (Schug and Gottlieb, 2009; Sapandowski et al., 2015).

Overall, these observations indicate that our algorithm successfully identifies several metabolites and genes that may be targeted for cancer treatment. The observations in both MMNs and RRNs show the necessity for combining both topology- and objective-dependent analyses in identification of suitable therapeutic targets. These approaches may be employed together with other omics technologies, to identify and develop targeted therapeutic strategies in other cancers.

## Author contributions

GB and AM conceptualization. GB methodology. GB, RB, and MA formal analysis. GB and RB data curation. EE and MNK experimental validation. GB and RB visualization. GB, RB, EE, and MNK writing initial draft. All authors final draft. AM supervision.

### Conflict of interest statement

The authors declare that the research was conducted in the absence of any commercial or financial relationships that could be construed as a potential conflict of interest.
