# Supplementary material for: Metabolic Network-Based Identification and Prioritization of Anticancer Targets Based on Expression Data in Hepatocellular Carcinoma
Source: Front Physiol. 2018 Jul 17;9:916. doi: 10.3389/fphys.2018.00916 (PMC6056771; doi:10.3389/fphys.2018.00916)
Supplement: Supplementary file 2 [file Image_1.PDF]

Supplementary Material

Metabolic network-based identification and prioritization of anticancer targets based on expression data: a hepatocellular carcinoma study

Gholamreza Bidkhori[1¶], Rui Benfeitas[1¶], Ezgi Elmas[1], Meisam Naeimi Kararoudi[1], Muhammad Arif[1], Mathias Uhlen[1], Jens Nielsen[2], Adil Mardinoglu[1,2,*]

[1] Science for Life Laboratory, KTH - Royal Institute of Technology, SE-171 21, Stockholm, Sweden,

[2] Department of Biology and Biological Engineering, Chalmers University of Technology, Gothenburg, Sweden.

[¶] These authors contributed equally.

***Correspondence:** Adil Mardinoglu: adilm@scilifelab.se

This file contains:

Captions for Supplementary Files 1 – 5

Supplementary Figs. 1 and 2

Supplementary File captions:

Supplementary File 1. Metabolic task file.

Supplementary File 2. Clone IDs and primer sequences for HepG2 and Hep3B cell line experiments.

Supplementary File 3. 50 personalized HCC and noncancerous GEMs as well as reference GEM.

Supplementary File 4. Antimetabolite, controlling, topology parameters and dispensability in MMN. HCC-exclusive antimetabolites, and controlling metabolites. Antimetabolites exclude pool metabolites, and antimetabolites simultaneously identified in HCC and noncancerous GEMs.

Supplementary File 5. *In silico* silencing, controlling, topology parameters and dispensability in RRN. HCC-exclusive gene targets determined based on *in silico* gene silencing, and controlling genes. This list excludes those genes simultaneously identified in HCC and noncancerous GEMs.

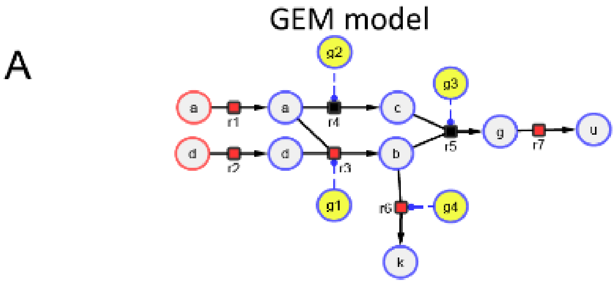

S matrix

	r1	r2	r3	r4	r5	r6	r7
a	1	0	-1	-1	0	0	0
b	0	0	1	0	-1	-1	0
g	0	0	0	0	1	0	-1
d_out	0	-1	0	0	0	0	0
a_out	-1	0	0	0	0	0	0
d	0	1	-1	0	0	0	0
c	0	0	0	1	-1	0	0
k	0	0	0	0	0	1	0
u	0	0	0	0	0	0	1

rev vector

rev	0	0	0	1	1	0	1

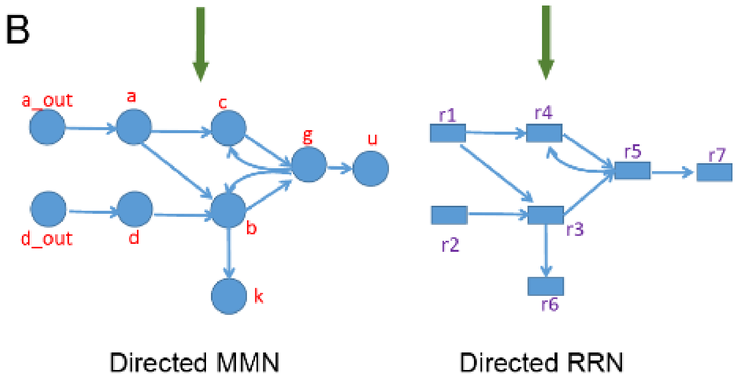

Supplementary Fig. 1. Data model and corresponding MMN and RRN. A toy model with 7 metabolites, 4 genes (*g1* to *g4*), and 9 reactions (*r1* to *r9*) including 2 exchange reactions is converted to directed MMN and RRN. This is accomplished through each model's S matrix and rev vector. Reaction *r7* is considered as objective function, and *r4* and *r5* are reversible.

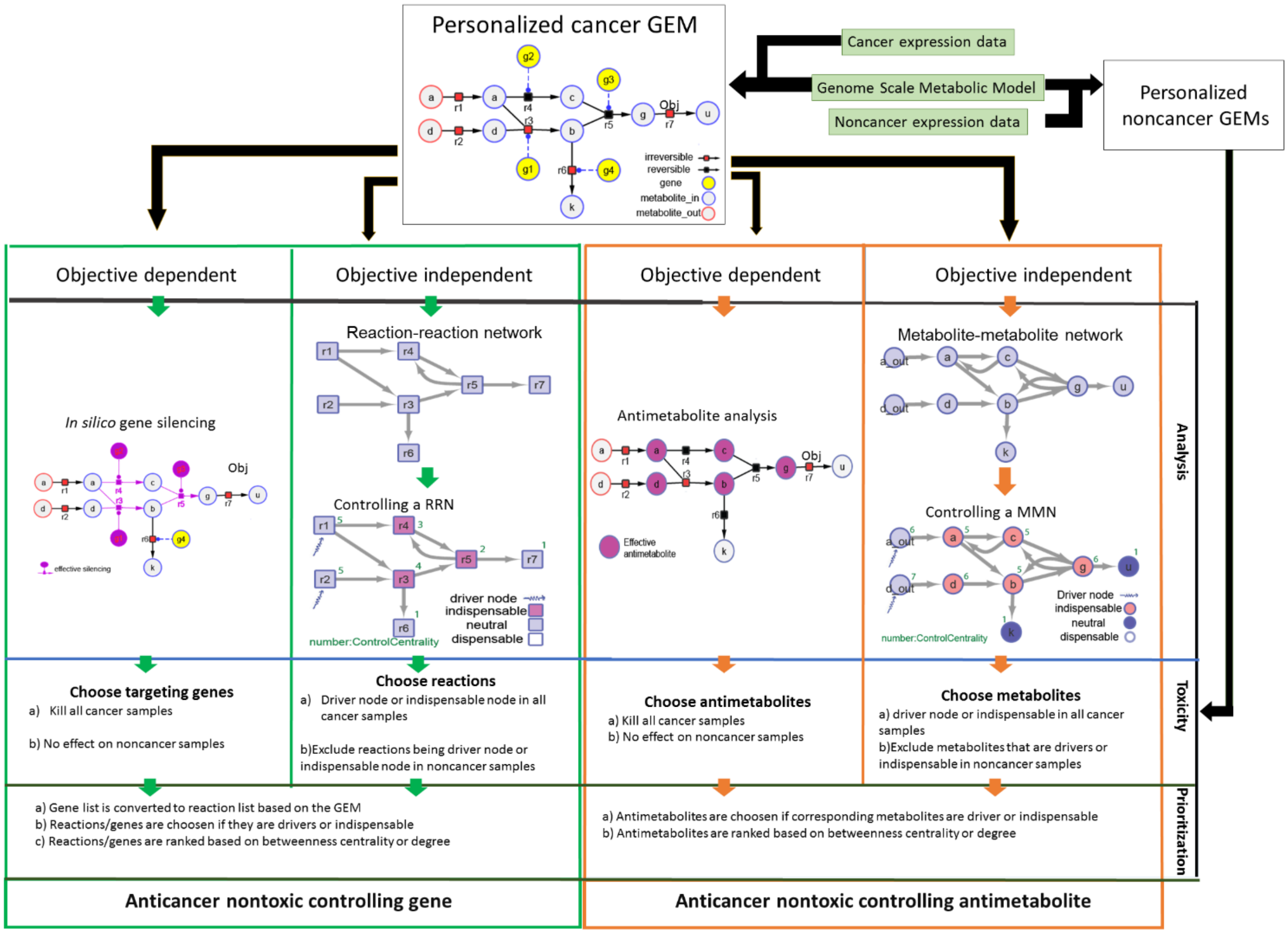

Supplementary Fig. 2 – Full algorithm description. We developed a method for anticancer prioritization by employing metabolic network analysis. Personalized GEMs are converted to MMN and RRN. Analysis of these networks employs both objective dependent (i.e. flux-balance analysis based methods), and objective independent (controllability, centrality, and dispensability). We then combine the predictions of objective-dependent methods (antimetabolite analysis and lethality prediction for gene silencing), with those of objective-independent methods (minimum driver set nodes, indispensable, and central nodes), we obtain a ranked list of targetable nodes. At multiple steps, potential nodes are filtered out if they are also identified in noncancerous samples.
